# Market Formation in a Global Health Transition

**DOI:** 10.1016/j.eist.2021.05.003

**Published:** 2021-09

**Authors:** Freek de Haan, Ellen H.M. Moors, Arjen M. Dondorp, Wouter P.C. Boon

**Affiliations:** a Copernicus Institute of Sustainable Development, Utrecht University, Princetonlaan 8a, 3484 CB, Utrecht, the Netherlands; b Mahidol-Oxford Tropical Medicine Research Unit, Faculty of Tropical Medicine, Mahidol University, 73170 Bangkok, Thailand

**Keywords:** Market formation, Global innovation system, Technological innovation system, Structural couplings, Malaria, Drug resistance, Global health transition, Innovative therapies, Geography of innovation

## Abstract

Transition studies have started to focus on market formation in innovation systems. This article investigates market formation in a global health transition that was instigated by drug-resistant malaria. We explore how markets for Artemisinin-based Combination Therapies (ACT) in the Greater Mekong Subregion (GMS) were formed at multiple geographical scales and locations. The study reveals the role of public institutes, academia and partnerships in early innovation system development. It demonstrates how transnational organizations created a supportive global landscape for ACT development and deployment. It then reveals how these advancements led to the formation of public-sector and private-sector ACT markets in the GMS. We illustrate how market formation activities took place on global, national and local scales and how structural couplings enabled the functioning of this global innovation system. The lessons learned are particularly relevant now that drug-resistant malaria has once more emerged in the GMS, urgently calling for new therapies and associated end-user markets.

## 1 Introduction

Malaria is a poverty-related infectious disease caused by plasmodium parasites. Although the global malaria burden has been significantly reduced in the last decades, the disease still takes nearly half a million lives a year (WHO, 2018). One important contributor to the reduced malaria burden has been the global transition from conventional monotherapies to Artemisinin-based Combination Therapies (ACT). The transition to this radical new treatment regime took off at the end of the previous millennium when parasites in the Greater Mekong Subregion (GMS) in Southeast Asia had become resistant to all then-conventional mono-therapies. As a result, malaria had become difficult to treat in the region and new therapies were urgently required. The situation even worsened when multidrug resistance spread further to India and Africa, resulting in dramatic increases in global malaria morbidity and mortality (Packard, 2014; Trape, 2001). However, despite this emerging health crisis the global uptake of ACT was slow and for years, patients were treated with outdated, substandard or even counterfeit medicines (Bosman and Mendis, 2007; Dondorp et al., 2004; Newton et al., 2006; O’Connell et al., 2011). Collective efforts at all levels of the antimalarial value chain were needed for the transition to ACT to unfold, e.g. at the level of drug development, diffusion and actual deployment (Yadav, 2009). Moreover, the formation of markets for this life-saving class of therapies required the establishment of new institutional, regulatory and financial arrangements.

This article analyzes what factors delayed the transition from conventional monotherapies to ACT and which activities at the global, national and local scales eventually enabled the shift. We particularly focus on the formation of ACT markets in the GMS, the global epicenter of drug-resistant malaria. The *innovation systems* framework is applied to understand the unfolding of this socio-technological transition. This framework claims that transitions do not occur in isolation but rather as a result of interactions between actor networks and institutions that are involved in the generation, diffusion and utilization of technologies (Edquist, 1997).

Investigating the transition from conventional monotherapies to ACT in the GMS emphasizes two important characteristics of the innovation systems framework. First, research, development, and deployment of antimalarial therapies take place at different geographical scales and in various countries. As we will demonstrate in our case, knowledge and production centered mostly in China and at the Thai-Myanmar border, while the market-creating activities occurred within the individual GMS countries. Transnational coordination was performed by globally operating institutes, including the World Health Organization (WHO), the Global Fund to fight Aids, Tuberculosis and Malaria (GFATM), Drugs for Neglected Diseases initiative (DNDi), and the Medicines for Malaria Venture (MMV). This incites crucial questions about how these different geographical scales and corresponding actor groups align and interact. With these questions we build on the recent interest in Global Innovation Systems (Binz and Truffer, 2017), which calls for research into spatially open, multi-scalar systems that are linked through structural couplings.

Second, the uptake of innovative therapies for malaria and other poverty-related infectious diseases has been characterized as slow and challenging, even when drugs are clinically superior to failing alternatives (Bosman and Mendis, 2007; O’Connell et al., 2011; Sheikh and Uplekar, 2016; Waning et al., 2010). Challenges have, amongst others, been associated with uncoordinated stakeholders, misalignment with institutional frameworks, and deficient health systems (Arrow et al., 2004; Sunyoto et al., 2019; Williams et al., 2004; Yadav, 2009). A more conceptual and empirical understanding of market formation in transitions can provide guidance in addressing these types of innovation challenges (Boon et al., 2020). A new line of research focuses on the processes through which markets emerge and evolve in innovation systems (Dewald and Truffer, 2012, 2011; Ottosson et al., 2020). This conceptualization of market formation has proven to be useful for analyzing transitions in the medical domain by providing insights into health-related institutional complexities (Kukk et al., 2016; Moors et al., 2018). In the present study, market formation is multifaceted as countries and localities in the GMS display different epidemiological contexts and health system structures (Cui et al., 2012; WHO, 2017a). At the same time, the formation of markets for innovative antimalarial therapies cannot be separated from global coordination and developments.

Building on these two points, this article aims to understand how ACT markets in the GMS have been formed at multiple geographical scales and locations. Our conceptual framework enables to take into account *all* system components rather than only those at the very end of the supply chain (Dewald and Truffer, 2011; Moors et al., 2018). Doing so, the research theoretically adds to innovation system literature, emphasizing the dynamics of system development and industry emergence across different geographical scales and locations (Binz and Truffer, 2017; Coenen et al., 2012) with a focus on market formation in a global health transition. This also contributes to the much-needed understanding of science, technology and innovation dynamics in times of global health emergencies.

An important practical contribution relates to the current epidemiological situation in the GMS. It is time to learn lessons from the market formation of ACT because the region is on the verge of yet another antimalarial drug transition. The era of ACT is coming to an end now that malaria parasites in the GMS have started to develop resistance to artemisinin and partner drug combinations (Amar-atunga et al., 2016; Dondorp et al., 2009). As a result, efficacy of ACT is declining and a transition to new and more sustainable therapies is once more required (van der Pluijm et al., 2021). The current situation in the GMS, with over 50% treatment failure in some areas, does not permit another slow drug transition because this would inevitably put further pressure on ACT efficacy and increase the risk of resistance spreading to Africa. The latter scenario could have major public health implications, both in terms of clinical outcomes and economic burden (Lubell et al., 2014) and could reverse all gains that have been made against the disease (Menard and Dondorp, 2017). Hence, the article aims to learn lessons from the transition to ACT to facilitate the formation of markets for next-generation antimalarial therapies. These lessons also apply to other endemic regions and to other poverty-related infectious diseases such as tuberculosis and HIV, which confront populations and governments with similar challenges.

Section 2 elaborates on our theoretical approach on the formation of markets at multiple geographical scales and locations and Section 3 gives an overview of the methods used. Section 4 presents the results, while Section 5 provides a discussion of the findings, including the practical implications for future therapies and Section 6 gives the conclusions of the study.

**The battle against drug-resistant malaria in the Greater Mekong Subregion**

Malaria continues to be a global health challenge of significant proportions. Each year, over 200 million people are infected and nearly half a million die because of malaria (WHO, 2018). The majority of the malaria burden is situated in Sub-Saharan Africa, where mainly children under five years of age are at risk. This is because those young children have not yet acquired any form of immunity against the disease, in contrast to most adults in high endemic settings. In lower transmission settings, including in the Greater Mekong Subregion (GMS), malaria is less of a pediatric disease and rather affects adults, often forest workers and migrant populations in remote border areas (McMichael and Healy, 2017).

**Fig. 1:**
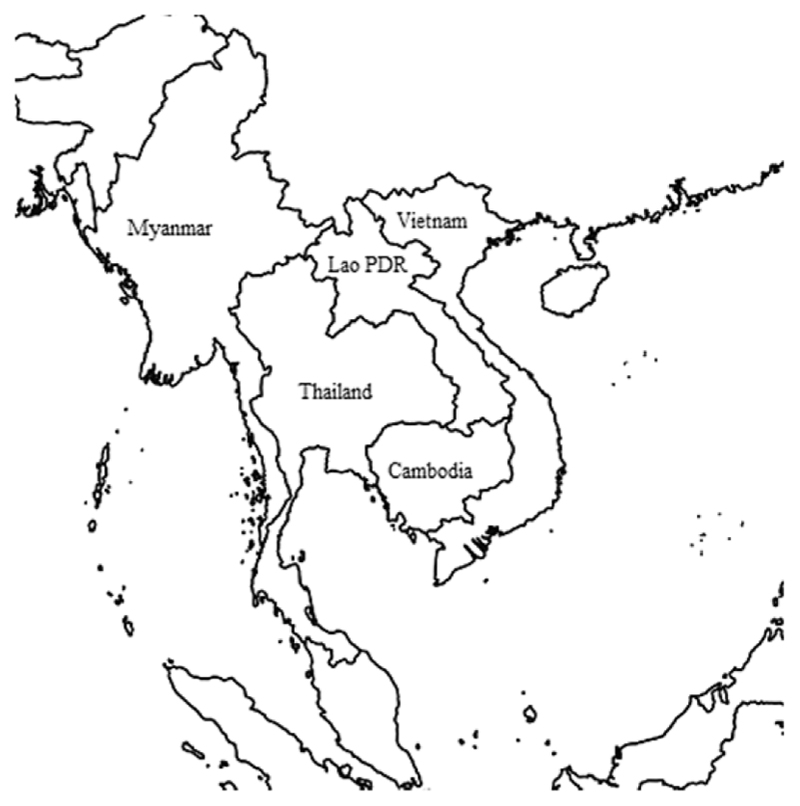
Map of the Greater Mekong Subregion.

The GMS is a region in South East Asia that is connected through the Mekong river and incorporates five countries (Fig. 1): Thailand, Cambodia, Myanmar, Vietnam and Lao PDR. All five countries are represented by their own health system contexts, political structures and malaria dynamics (Cui et al., 2012). With the exemption of Thailand (an upper-middle income economy), the GMS countries are all classified as lower-middle income economies (Worldbank, 2020). Significant improvements have been made in the battle against malaria in the GMS with a 74% incidence reduction between 2012 and 2017 (WHO, 2017b). A number of strategies have been associated with these achievements, including improved surveillance, mosquito control and the increased availability of diagnostic tools and effective drugs (Cibulskis et al., 2016). Despite the historic low burden, the GMS remains a focus area for malaria researchers and policy makers. This is because resistance to antimalarial therapies has repeatedly emerged in the GMS, including to chloroquine and sulfadoxine-pyrimethamine, the most frequently used antimalarial therapies in the pre-artemisinin era (Packard, 2014).

The ability of malaria parasites to acquire drug resistance is a consequence of genetics and natural selection, reinforced by their short lifecycle and rapid reproduction (Klein, 2013). When medication is administered to a patient in inadequate dose or form, some parasites may survive the treatment regime. Those parasites then have an evolutionary advantage over drug susceptible parasites and may become dominant in the parasite population. The reason that this has repeatedly occurred in the GMS is because there is limited genetic diversity amongst parasites in this low transmission area. Moreover, inadequate deployment practices are likely to have contributed to the repeated emergence and spread of drug resistance in the GMS region (Klein, 2013; White, 1999). To mitigate the risk of future occasions of antimalarial drug resistance, it is therefore important that drug regimens eliminate *all* the parasites in the bloodstream. Deployment of subtherapeutic doses, low-quality medicines, or premature finalization of treatment courses should be avoided at all times (WHO, 2006).

## 2 Theoretical approach

In order to understand how ACT markets were created under the pressure of drug resistance, we first elaborate on pharmaceutical innovation for poverty-related infectious diseases, requiring activities that are dispersed yet coordinated (Section 2.1). We then introduce the Global Innovation Systems (GIS) framework as a perspective to deal with the geographical dispersion of innovation system components and the interlinking structural couplings (Section 2.2). Finally, we zoom in on the formation of markets for innovative therapies in the context of multi-scalar innovation systems (Section 2.3).

### 2.1 Pharmaceutical innovation for poverty-related infectious diseases: the need for a multi-scalar innovation system concept

**Fig. 2:**
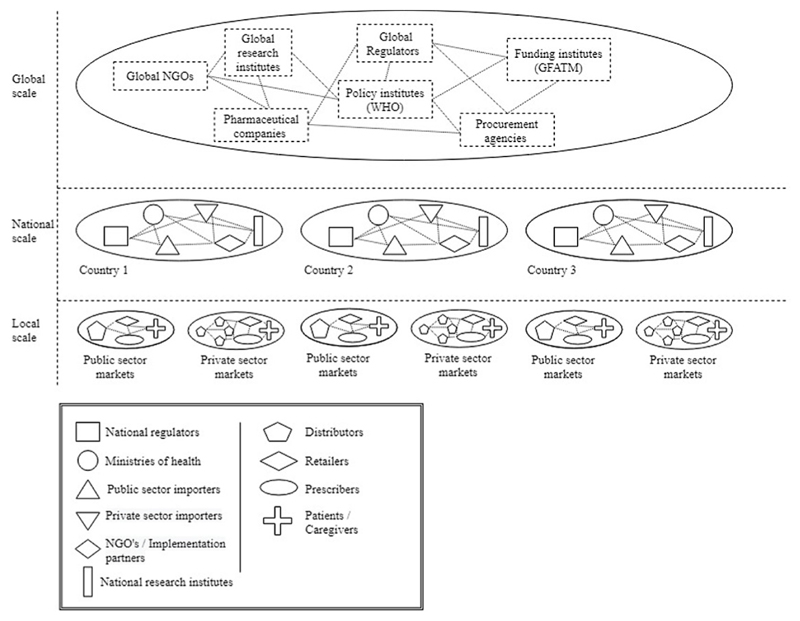
Key actors in pharmaceutical innovation for poverty-related infectious diseases at the global, national and local scales.

To address the threat of drug resistance for poverty-related infectious diseases, transitions to more sustainable drug regimens are required. This implies that such regimens need to be discovered, developed, integrated in distribution chains and adopted by prescribers and patients (Moors et al., 2014; Tindana et al., 2021; Yadav, 2015). These innovation processes involve multiple actors, networks and institutions on different geographical scales and locations, as is depicted in Fig. 2. When perceiving transition activities as being distributed yet coordinated, the innovation systems framework is helpful for analysis.

Pharmaceutical research and development have traditionally been dominated by multinational organizations that collaborate with research institutes across the globe. However, pharmaceutical companies have for long ignored diseases of the poor because of lacking commercial incentives (Muñoz et al., 2015). In response, attention has shifted to alternative drug development models, including governmental research projects and product-development partnerships (Bompart et al., 2011; Hoogstraaten et al., 2020; Kitchen et al., 2006; Ubben and Poll, 2013). Globally-operating institutes such as the World Health Organization (WHO) and the Global Fund to fight Aids, Tuberculosis and Malaria (GFATM) regulate the global market place for poverty-related infectious diseases (’t Hoen et al., 2014; Arrow et al., 2004). Their authorization and support are considered a pre-requisite for safe deployment and for eligibility to institutional arrangements such as inclusion in normative guidelines and global subsidy programs. These arrangements then influence country-level market formation activities in endemic countries, including product registration and adoption in national treatment guidelines (Sheikh and Uplekar, 2016; Williams et al., 2004).

Once a new therapy is approved and registered at the country-level, implementation programs can be organized by Ministries of Health (MoH) to integrate the innovative therapy into distribution channels. Public sector distribution channels are top-down coordinated by national governments, while private sector distribution channels are generally more diverse, unsupervised and commercials in nature (Palafox et al., 2014; Yadav, 2015). At the end of the distribution channels, the innovative therapy is delivered at local-scale prescribers and retailers, who are responsible for administering the therapy to patients and their caregivers. Patients need to consume drugs in a rational way to achieve optimal clinical results and to mitigate the risk of drug resistance (Klein, 2013).

What becomes clear is that development, regulation, distribution and utilization of innovative therapies for poverty-related infectious diseases takes place at different geographical scales (global, national and local) and locations (Fig. 2). If we want to investigate drug transitions for poverty-related diseases with a specific interest in market formation, then we need a conceptualization of innovation systems that transcends geographical scales and borders.

### 2.2 Innovation systems at multiple geographical scales and locations

The innovation systems framework departs from the premise that innovation is a collective effort that involves many actor groups and institutional frameworks which are organized in a complex social system (Edquist, 1997). Innovation systems have originally been delineated by a regional, sectoral or national scope. More recently, the technological innovation system framework was introduced to analyze the emergence and societal embedding of specific technologies. The performance of technological innovation systems can be evaluated by assessing a set of seven system functions. In joint interaction, these system functions contribute to a favorable environment for emerging technology (Bergek et al., 2008; Hekkert et al., 2007). The seven system functions as identified and described in-depth by Hekkert et al. (2007) plus their interpretation in terms of innovative therapies are summarized in Table 1.

Traditional innovation system approaches do not explain the effects of geographical dispersion of system components and the transnational nature of innovation dynamics (Coenen and Truffer, 2012). To address these lacunae, Binz and Truffer (2017) introduced the notion of Global Innovation Systems (GIS). The GIS perspective offers a framework for analyzing innovation processes in transnational contexts by focusing on the generation of resources in multi-scalar subsystems and the formation of couplings between the geographically-dispersed subsystems.

The GIS framework integrates the key system functions of technological innovation systems. Binz and Truffer (2017) conceptualize the functions as system resources and particularly focus on knowledge creation, resource mobilization, market formation and legitimation processes. They add a geographical dimension by arguing that systems can be organized on multiple geographical locations (Bergek et al., 2015; Coenen and Truffer, 2012). These multi-located systems are made effective through the organization of so-called *structural couplings*. Structural couplings are actors, networks or institutions spanning across or overlapping between various innovation subsystems. Structural couplings can, for example, be transnationally-operating institutions, international associations and NGOs. Fig. 2 illustrates that pharmaceutical innovation in global health transitions is a multi-scalar endeavor. In this article, we are interested in understanding how structural couplings [SC] are attained between geographical scales and locations and how this contributes to the performance of the innovation system in the context of a global health transition.

### 2.3 Market formation in multi-scalar innovation systems

Market formation is one of the technological innovation system functions (Bergek et al., 2008; Hekkert et al., 2007) and refers to the activities that directly contribute to the availability and accessibility of the innovative therapy by potential end-users (Table 1). In line with technological innovation systems literature, we emphasize the interplay of market formation with the other system functions. For example, legitimacy creation for an emerging therapy can affect how market boundaries are defined, while knowledge development on safety and efficacy of medical technologies dictates its target patient group.

In this article, we build on the work of Dewald & Truffer (2011, 2012) who have studied market formation as a system function in more depth. According to them, markets are pivotal to the long-term success of innovative technologies and a more encompassing view of market formation is required. In order to arrive at such improved understanding, they introduce three market formation sub-processes [MF1–3] (Table 1). Given the potentially high divergence in market developments, Dewald and Truffer (2011) propose the formation of *market segmentations* [MF1] and their interactions as the first subprocess of market formation. The term market segments refers to the innovation system substructures that are oriented at specific product categories or end-user groups and include all related actors, networks and institutions.

In subsequent work, Dewald and Truffer (2012) claim that there is an important lack of attention to economic geography in market formation analysis. To address this spatial dimension, they provide a more elaborate conceptual framework by adding two subprocesses. The formation of *market transactions* [MF2] refers to the establishment of an exchange relationships between suppliers and customers. *End-user profiles* [MF3] relates to the active role of users and consumers by developing preference structures and utilization practices.

In line with Dewald and Truffer (2012), we define market formation as: the demarcation of markets and target groups [MF1], the creation of transactions [MF2], and the way in which users participate in the innovation process [MF3]. Following the related conceptualization of three critical processes by Ottosson et al. (2020), we conceptualize market formation as being in continuous interaction with the other system functions. As depicted in Fig. 2, end-user markets for innovative therapies reside at the local scale but they are determined by (and sometimes are determining) activities and structures at the national and global scales. We add a multi-scalar dimension to our conceptualization by investigating how structural couplings between geographical dispersed innovation system components contribute to market formation in a global health transition.

## 3 Methods

### 3.1 Case selection and delineation

In order to analyze how markets are formed at multiple geographical scales and locations, we explored the transition from conventional monotherapies to Artemisinin-based Combination Therapies (ACT). ACT comprise all antimalarial therapies that combine an artemisinin compound with a slower working partner drug (White, 1999). The GMS was selected as spatial delineation of the study because this region is considered to be the global epicenter of drug-resistant malaria (Cui et al., 2012). We followed this drug transition from the discovery of artemisinin in the early 1970s, until the completion of forming ACT markets in the GMS in the mid-2010s.

In line with the innovation system framework, we took a holistic perspective to understand the market formation of ACT in this global health transition. We considered ACT as a new generation of technologies around which a technological innovation system has emerged and evolved on different geographical scales and locations. Our analysis followed the transition to ACT from the early stages of drug development, regulation and industry creation, until the establishment of distribution networks and prescription practices.

### 3.2 Data collection

An event history analysis was selected as research design to investigate how ACT markets were created in the GMS. This research design is considered a suitable approach for studying technological transitions that involve a wide array of actor networks and institutions (Wieczorek and Hekkert, 2012). As a first step, the actors, networks and institutions of the ACT innovation system were identified and mapped. Then, a timeline was constructed which included events that represented the transition from conventional monotherapies to ACT, with an emphasis on market formation in the GMS. Data were collected through literature review and expert interviews with respondents from different stakeholder groups.

The literature review comprised the collection and analysis of both peer-reviewed and gray literature. Peer-reviewed literature was accessed through the search engines Web of Science and Google Scholar, using broad defined search queries that aimed to retrieve all literature subjected to the transition to ACT. An example of such a query was: (“artemisinin” or “artemether” or “artesunate” or “dihydroartemisinin”) or (“ACT” and “malaria”) and (“implementation” or “transition” or “introduction” or “innovation” or “market”). Gray literature was accessed through using similar search terminologies in the search engine Google, and by performing targeted searches at institutional websites such as the WHO,^1^ GFATM,^2^ MSF,^3^ USAID,^4^ MMV^5^ and DNDi.^6^ Selected gray literature included policy reports, institutional press releases, and research articles at websites of global health organizations.

The main goal of the literature review was to identify the key events that affected the transition from conventional monotherapies to ACT with an emphasis on the formation of markets in the GMS. Therefore, the titles, summaries and abstracts of the obtained literature were examined. If the document was considered relevant for the purpose of the study, the full content was assessed. Events that either positively or negatively influenced the transition to ACT in the GMS plus the corresponding dates were extracted from the selected literature and listed in a database. The same literature was also used to gain contextual insights beyond the mere identification of events. For all selected articles, forward and backward citation checks were done to identify additional data sources. Eventually, the dataset was used to construct a preliminary timeline, which included a total of 85 events. To validate the timeline and complement it with additional insights, six expert interviews were conducted. For these interviews, professionals active in organizations that have been involved in the transition to ACT were approached. Table 2 provides the list of interview respondents.

Interviews followed a semi-structured protocol in which topics were selected based on the affiliation and expertise of the respondent. We asked general questions about the stakeholders, networks and institutions that had been involved in the global transition from conventional monotherapies to ACT. Furthermore, the respondents were asked to identify and confirm events that led to the formation of ACT markets in the GMS and to provide contextual background to these events. Each interview took between 35 and 81 min and was audio recorded with consent from the interviewee. Anonymity was granted to all respondents.

### 3.3 Data analysis

Based on the literature review and expert interviews, a final event timeline was constructed. Then, the events were subjected to a process of coding. Each event was allocated to one of the theoretical constructs as described in Table 1 by using the operationalized indicators in Table 3. Codes were attributed to each event and included in the database.

The coded events were discussed amongst the researchers for verification purposes. Differences in interpretations were followed-up by discussion until consensus was reached. Based on the coded timeline of events, the corresponding dates, and the contextual comments, a narrative was constructed and a content analysis of the formation of ACT markets in the GMS was performed. The narrative followed the three major episodes of market formation that had emerged from the collected data. We then plotted diagrams to distinguish actors and functional dynamics at local, national and global scales, as well as the relations between them. These plots enabled us to identify structural couplings [SC] between the geographically dispersed subsystems, using the definition that we provided in Section 2.2 as a heuristic. Finally, a case interpretation was written for each episode, in which we analyze the multi-scalar transition dynamics. Appendix A provides more detailed information on the research methodology of the study.

## 4 Results

Three major episodes of the formation of ACT markets in the GMS were identified. Section 4.1 begins with a narrative outline of the discovery of ACT and the early evolution of the ACT innovation system from the early 1970s onwards (Episode 1). Section 4.2 reveals how a supportive global landscape for ACT production and distribution was created through collective efforts by transnational institutes and organizations (Episode 2). Section 4.3 demonstrates how these advancements led to the formation of public-sector and private-sector markets for ACT in the GMS (Episode 3). Each episode ends with an analysis of the multi-scalar transition dynamics and the market formation processes.

### 4.1 Episode 1: the early evolution of the ACT innovation system (early 1970s to late 1990s)

#### 4.1.1 Historical overview

**Fig. 3:**
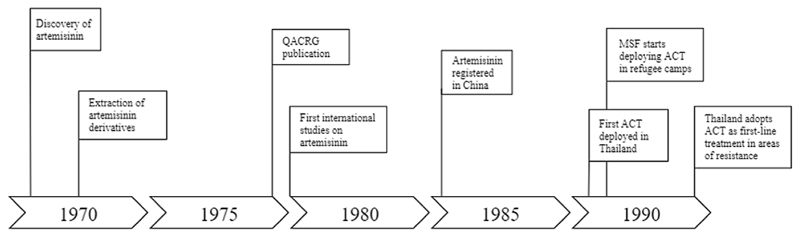
Timeline of the early evolution of the ACT innovation system (Episode 1).

The discovery of the antimalarial potential of artemisinin can be traced back to the late 1960s. By then, Vietnamese troops were suffering heavily from malaria during the war with the United States, and drug resistance further amplified the disease burden. Because of the limited in-country capability for drug development, the Vietnamese government decided to approach neighboring China with a call for support in 1967 (Faurant, 2011). China met the request with the establishment of ‘Project 523′, a governmental research program in which more than 500 researchers engaged in the search for new antimalarial drug compounds (Cui and Su, 2009; Faurant, 2011). Within Project 523, a large number of drug candidates were identified and screened, including over 200 plants that had been used in traditional Chinese medicine (Cui and Su, 2009; Liao, 2009; Tu, 2011). One of those plants, *Artemisia annua*, demonstrated promising antimalarial potential in early assessments. Follow-up studies soon confirmed the efficacy of *Artemisia annua,* and in 1972, researchers from the Academic of Military Medical Sciences, led by professor Youyou Tu, managed to extract the active ingredient of the plant (Tu, 2011). In the following years, the extraction process was further optimized, which resulted in three highly effective artemisinin derivatives that would later become the backbone of ACT therapies: artesunate, artemether, and dihydroartemisinin. Years later, in 2015, professor Youyou Tu would be awarded a Nobel prize for her role in the discovery of artemisinin and its derivatives.

Due to the cultural and political situation in China, publishing medical research in international journals was not a common practice. The few written articles that were subjected to the discovery of artemisinin and its derivatives were exclusively reported in Chinese and were hardly picked up by the rest of the world (Chang, 2016; Su and Miller, 2015). It would take until 1979 before the first article was published in English, by the Qinghaosu Antimalaria Coordinating Research Group (QACRG). In this paper, the chemical structure and pharmacological properties of the artemisinin derivatives were discussed based on a number of in-vitro studies, animal models, and clinical studies with over 2000 Chinese malaria patients (QACRG, 1979). The QACRG publication drew attention from international clinicians and researchers because by then, drug-resistant malaria had become widely acknowledged as a serious global health threat (Chang, 2016). In 1985, artemisinin was registered as a new drug compound in China, but in line with prevailing Chinese practices, no patents were requested or filed for artemisinin and its derivatives (Guo, 2016).

In successive clinical studies in China and the GMS countries, the artemisinin derivatives further proved to be safe and extremely potent in eliminating malaria parasites (Hien and White, 1993; Tu, 2011). Over twenty studies confirmed the safety and efficacy of the artemisinin derivatives between 1979 and 1992 (e.g. Hien and White, 1993; Jiang et al., 1982; Li et al., 1984). Moreover, in 1991, the Ministries of Health (MoH) of both China and Vietnam started to distribute artemisinin monotherapies to malaria patients within their borders (WHO, 1998). Initially, only a few thousand doses were disseminated in both countries, but this number substantially grew in the following years.

Although the diffusion of artemisinin in Asia was generating momentum, one significant problem remained. The short plasma half-life of the artemisinin derivatives was associated with high risk of returning infections (recrudescence) and an accelerated risk of artemisinin resistance when used as a monotherapy (White, 1999). Therefore, researchers started to advocate the exclusive deployment of artemisinin derivatives in combination with a longer-acting partner drug, creating Artemisinin-based Combination Therapies (ACT). Thereby, recrudescence would be averted and the risk of artemisinin resistance would be reduced (Nosten and Brasseur, 2002). Artesunate, co-administered with mefloquine was first deployed in a hospital setting in Thailand in 1991 and the results of this clinical study were closely monitored. The resulting insights demonstrated that the combination was a highly effective one, and indeed reduced the risk of recrudescence (Looareesuwan et al., 1992). In the second half of 1991, a similar combination was deployed on a larger scale in a refugee camp at the Thai-Myanmar border under coordination of Médecins Sans Frontières (MSF), a globally operating humanitarian organization that was involved in the provision of medical aid in this politically unstable region (Luxemburger et al., 1994). Again, the clinical results were monitored and a consistent over 95% cure rate was found, which prompted MSF to adopt this combination as the standard antimalarial treatment in refugee camps along the Thai-Myanmar border (Price et al., 1995).

In the following years, more and more studies confirmed the efficacy and safety of this co-administered therapy within different Asian settings (Luxemburger et al., 1994). Without exception, the superiority of ACT compared to conventional monotherapies was demonstrated, both in terms of clinical outcome and recrudescence rates. Different dose regimens were explored and the cumulative insights of these studies suggested a 3-day oral intake as optimal (White et al., 2015). In 1995, the Thai government accepted ACT and adopted co-administered artesunate plus mefloquine as first-line therapy in all areas where resistance to conventional antimalarial monotherapies had been observed (ACTwatch, 2016a).

#### 4.1.2 Analysis of episode 1

**Fig. 4:**
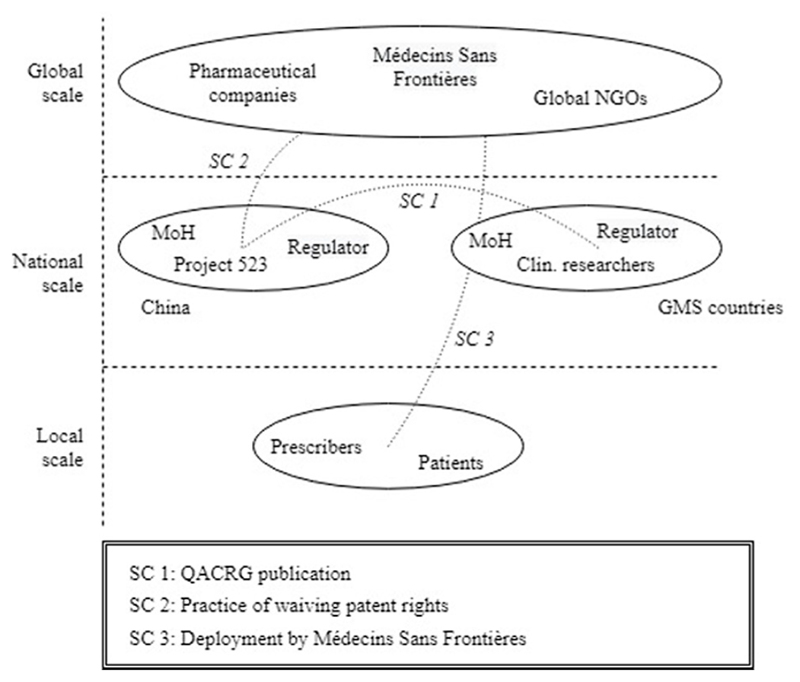
Emerging structural couplings in Episode 1.

The first episode revealed how the pressure of multidrug resistant malaria led to the emergence and early evolution of the ACT innovation system. The narrative demonstrated that the boundaries of the ACT innovation system expanded from China, where artemisinin was discovered, to the GMS countries, where ACT deployment was further advanced. We observe how three structural couplings [SC1-SC3] between geographically dispersed subsystems appeared in this episode, which enabled the functioning of the ACT innovation system and its expansion along geographical scales and locations (Fig. 4).

A first structural coupling [SC1] was attained between China and the GMS countries through the QACRG publication that exposed the antimalarial potential of artemisinin to the international community. Prior to this, the establishment of Project 523 [F1] had resulted in the discovery of artemisinin and its derivatives [F4], and had provided the first clinical evidence of its efficacy and safety [F2]. However, despite the promising potential of artemisinin, the acquired knowledge was only diffused beyond the Chinese border after the QACRG publication [F3]. This publication opened-up the antimalarial capacity of artemisinin to the rest of the world and coupled the Chinese discovery of artemisinin with the clinicians in the GMS countries that were in urgent need of solutions to treat drug-resistant malaria.

What we observe upon the attainment of this first structural coupling is that a reinforcing pattern, or virtuous cycle, emerges between clinical studies with artemisinin in the GMS [F2, F3], and the cumulative knowledge of these studies which collectively guided towards the optimal dose-, and prescription form of artemisinin and later ACT [F4]. This ensuing virtuous cycle also marks the first market formation activities for ACT in the GMS [F5]. Clinical researchers, who were struggling to treat drug-resistant malaria infections, engaged in the prescription of ACT. Doing so, they both offered ACT as a medical solution to their patients [F5 MF2] and simultaneously they contributed to the evidence-base [F2, F3] and optimal prescription form of ACT [F4, F5 MF3].

A second structural coupling [SC2], involving the global scale, appeared through the practice of waiving intellectual property rights by the research group that was responsible for the discovery of artemisinin. In contrast to common pharmaceutical innovation, no patents were requested for the artemisinin derivatives or the ACT that would later be introduced. This practice of waiving intellectual property rights would eventually enable both research-based and generic manufacturers to start ACT production [F1], leading to a viable industry, product competition and eventually to price reductions [F6] (see Episode 2).

A third structural coupling [SC3] was attained between the global and the local scales when Médecins Sans Frontières (MSF) started to engage in ACT deployment in local refugee camps at the Thai-Myanmar border. Until then, ACT was only deployed by local-scale clinicians and researchers, later endorsed by the Thai government [F7]. The involvement of MSF, a reputable global NGO, further contributed to legitimacy creation [F7], and to the uptake of ACT [F5 MF2] as first-line therapy in areas of drug resistance.

All three structural couplings that emerged in this episode contributed to the expansion of the ACT innovation system along geographical scales and locations. The next episode further elaborates on these developments and explains how a supportive global landscape for ACT development and deployment was created through collective efforts by transnationally operating organizations.

### 4.2 Episode 2: a supportive global landscape for ACT production and distribution (late 1990s to early 2010s)

#### 4.2.1 Historical overview

**Fig. 5:**
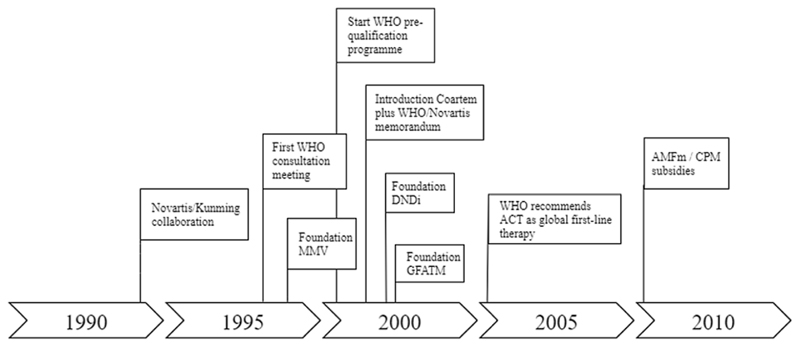
Timeline of a supportive global landscape for ACT production and distribution (Episode 2).

By the end of the 1990s, conventional monotherapies were failing throughout the world, leading to sharp increases in global malaria mortality and morbidity (WHO, 1998). In response to this emerging health crisis, the WHO organized a series of expert consultation meetings between 1998 and 2001. The goal of these meetings was to discuss the global impact of multidrug resistance and to develop policy accordingly (WHO, 2001, 1998). The WHO consultation meetings led to three guiding principles on how drug-resistant malaria ought to be approached. First, it was decided that artemisinin-based therapies would have to be deployed as first-line therapy in all areas where conventional monotherapies were failing. Second, artemisinin derivatives were to be used exclusively in combination with a partner drug. The prescription of artemisinin monotherapies had to be dissuaded in order to mitigate the risk of emerging artemisinin resistance. Third, it was stipulated that ACT should be deployed as *fixed-dosed* therapies. Contrary to co-administered medication, fixed-dosed therapies combine the individual drug compounds into one pill. This prescription form would encourage patients to comply to full drug regimens and to reduce the risk of cherry picking the highly effective artemisinin compound while rejecting the unpopular partner drug, which is often associated with nausea and other side effects (Interview 2). In 2006, the WHO came with a more universal statement regarding ACT deployment by recommending ACT as first-line therapy in *all* malaria endemic regions without any further restrictions (WHO, 2006). In that same year, the WHO also requested the pharmaceutical industry to withdraw the production and sale of oral artemisinin monotherapies. The goal of this request was to discourage the trade and the consumption of dangerous monotherapies and, in doing so, to mitigate the risk of artemisinin resistance (WHO, 2006). Most, but not all pharmaceutical companies adhered.

Meanwhile, significant supply-side challenges had to be overcome. Fixed-dose ACT was not yet commercially produced and thus not available for large-scale deployment. Moreover, the lengthy cultivation and extraction process of artemisinin contributed to a production price for ACT that was estimated to be 20–50 times higher than the average 0.10 USD for chloroquine (Arrow et al., 2004). This would confront governments and populations in malaria endemic settings with an insurmountable cost barrier (Interview 2).

Efforts by globally operating organizations and the initiation of a range of institutional frameworks eventually enabled overcoming these supply and cost challenges. A first major driver was the establishment of a partnership between the Swiss pharmaceutical company Novartis (by then still named Ciba-Geigy) and Kunming, a Chinese manufacturer of artemether (Spar and Delacey, 2008). Even ahead of the WHO recommendations, these organizations had already in 1994 joined forces in order to develop a fixed-dose combination of artemether-lumefantrine. In 2000 they successfully launched this drug for international use under the brand name Coartem®. Although Coartem® did not become the dominant therapy in most GMS countries, it has contributed to the global transition to ACT in several ways. Coartem® would become the first antimalarial drug added to the WHO list of essential medicines and to receive the WHO pre-qualification status, boosting its worldwide attention and eligibility to procurement subsidies. Initially established in 2001 during the HIV/AIDS pandemic, the WHO pre-qualification program supports resource-restricted countries with regulatory assessments to ensure that products meet global quality standards in a wide range of therapeutic areas, including malaria (’t Hoen et al., 2014) . Perhaps the most important contribution of the introduction of Coartem® has been the signing of an unprecedented memorandum. In 2001, the WHO and Novartis signed a ten-year contract, in which they agreed that Novartis would waive profits and instead sell Coartem® directly to the WHO at cost price. The WHO would then distribute the drugs to endemic countries which, in return, had to report three-month demand forecasts back to Novartis. This procurement system would enable Novartis to exponentially scale-up production of Coartem® without risking an unsold overproduction (Spar and Delacey, 2008).

It would take years after the introduction of Coartem® and CV8® (a fixed-dose combination of dihydroartemisinin-piperaquine offered on the internal market in Vietnam) for other fixed-dose ACT to be introduced. New product launches followed the initiation of two umbrella institutes that were founded to address the R&D gap for poverty-related diseases. The Medicines for Malaria Venture (MMV) was initiated by European governments and philanthropic organizations in 1999. A few years later, the Drugs for Neglected Disease initiative (DNDi) was founded for similar reasons by MSF and the WHO. Although DNDi had a broader product portfolio than just malaria, both organizations aimed to encourage partnerships between public and private organization in order to stimulate the development of medicines for poverty-related diseases, such as malaria. Since then, both MMV and DNDi have set-up and coordinated a number of product development partnerships which have led to the introduction of several fixed-dose ACT from 2007 onwards (Bompart et al., 2011; Ubben and Poll, 2013; Wells et al., 2013). In 2015, DNDi transferred all their malaria-related activities to MMV.

Patent rights were again waived for all the fixed-dose ACT that were introduced by the MMV and DNDi partnerships. As a result, generic producers were able to enter the market which eventually led to product competition and price reductions at the global market (Orsi et al., 2018). Moreover, similar contracts as the WHO/Novartis memorandum were signed for most of the newly introduced ACT combinations, which further enhanced their affordability and availability.

Despite all these efforts, the high production costs of artemisinin made ACT relatively expensive (Arrow et al., 2004). Malaria endemic countries would therefore still have to rely on external donor funds to be able to procure ACT (Interview 1 & 4). Initially, a number of small-scale subsidy programs supported endemic countries with the procurement of ACT. However, a true significant driver towards global affordability of ACT would be the foundation of the Global Fund to Fight Aids, Tuberculosis and Malaria (GFATM), a globally operating funding institute (Interview 4 & 6). Founded in January 2002, the GFATM now supports resource-restricted countries with procurement of expensive therapies through providing an ongoing and sustainable subsidy program. Since then, the GFATM has grown into a multi-billion operation that significantly contributes to affordability of ACT and many other therapies. In order to become eligible for GFATM donor subsidies, medicines need to be approved by a stringent regulatory authority such as EMA, FDA, or the WHO pre-qualification scheme (Orsi et al., 2018; Pelfrene et al., 2015). One critique to the initial GFATM subsidy was that it exclusively targeted public sectors while ignoring the private sector equivalents. In response to this critique, a private-sector subsidy was implemented by the GFATM in 2010, under the name Affordable Medicines Facility malaria (AFMm), and later scaled-up as Co-Payment Mechanism (CPM).

#### 4.2.2 Analysis of episode 2

**Fig. 6:**
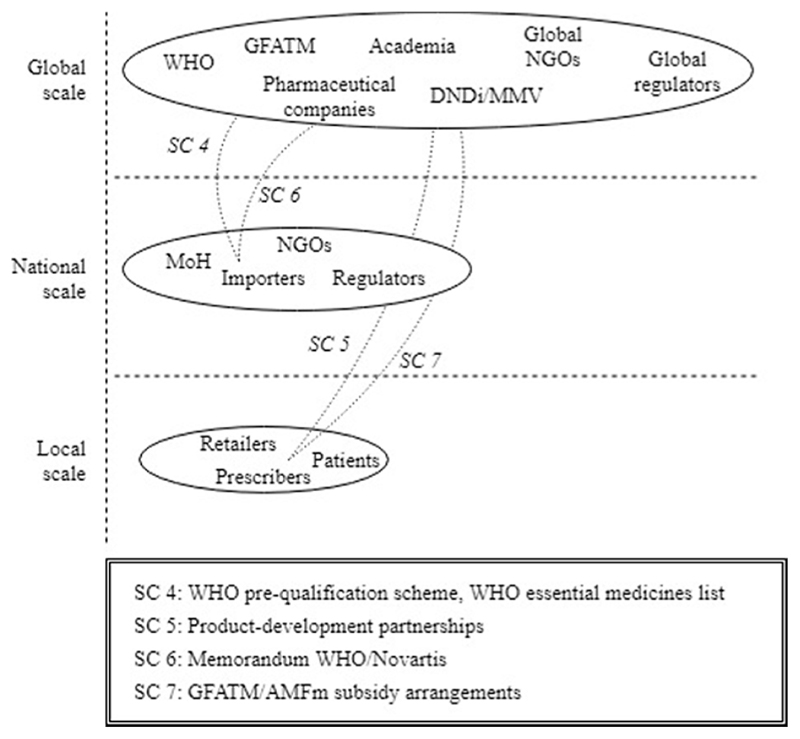
Emerging structural couplings in Episode 2.

The second episode revealed how the ACT innovation system further expanded from the GMS countries to the global and the local scales. We observed how a supportive global landscape for ACT development and deployment was created through collective efforts by transnational organizations including the WHO, GFATM, MMV, DNDi, and Novartis. Four structural couplings [SC4-SC7] were attained in the episode that have been critical to the functioning of the ACT innovation system along different geographical scales and locations (Fig. 6).

The first structural coupling [SC4] emerged between the global and national scale and comprised the WHO arrangements that supported national governments with the adoption of ACT. The WHO first synthesized and reviewed all clinical evidence in order to formulate the guiding principles on ACT deployment [F4] and then created legitimacy by recommending ACT as global first-line therapy [F7]. Additionally, the WHO established the regulatory pre-qualification scheme [F7] and the WHO essential medicines list [F4], which enabled countries to prioritize medical needs and to define the preconditions for safe deployment of innovative therapies. These WHO arrangements cleared the road for GMS governments to start engaging in ACT and they now contribute to a coordinated global marketplace for antimalarial therapies.

Nevertheless, two major barriers persisted towards the formation of end-user markets for ACTs. The first barrier was that fixed-dosed ACT was not yet commercially produced [F1], and so entrepreneurial activity was required to translate the medical-technical potential into actual end-products. The second barrier was that a transition to ACT would imply an insurmountable cost barrier for patients and governments in malaria endemic settings [F6]. Hence, financial resources were required to enhance ACT affordability. The narrative demonstrated how these barriers were addressed through attainment of three structural couplings.

The dearth of entrepreneurial activity was addressed in the form of product-development partnerships [SC5] that directly linked the global with the local scale. These product-development partnerships were initiated as instruments to stimulate innovation for malaria as a poverty-related disease and aimed to introduce ACT end-products that were ready for deployment. The first partnership was the collaboration between Novartis and Kunming which led to the introduction of Coartem® [F1]. Subsequently, several successful product-development partnerships were initiated by DNDi and MMV, which have further fueled the antimalarial pipeline with actual end-products [F1]. The fact that patent rights were waived for these ACTs enabled generic manufacturers to become active, stimulating further entrepreneurial activities [F1] and leading to a viable industry (see Episode 1, SC2).

Two other structural couplings contributed to overcoming financial resource barriers. One structural coupling [SC6], linking the global and national scales, emerged through the signing of the WHO/Novartis memorandum. In this memorandum, Novartis agreed with the WHO to waive profit margins and instead sell ACT at cost price [F6]. Not only did this reduce the price of this particular ACT [F6], it also set the standard for the ACT end-products that would later be introduced through the DNDi and MMV partnerships [F6]. The practice of waiving patent rights enabled generic manufacturers to enter the market, leading to further price reductions [F6].

A fourth structural coupling [SC7] emerged between the global and local scales through the GFATM and AMFm/CPM subsidy arrangements. Both these arrangements directly aimed to reduce the prices of ACT for malaria patients in endemic settings. The global-level pubic-sector GFATM [F5 MF1] and private-sector AMFm/CPM [F5 MF1] subsidies have been pivotal in mobilizing and channeling the necessary funds to improve affordability of ACT at the local levels [F6, F5 MF2]. The next episode explains how these advancements and country-level activities eventually resulted in the formation of actual end-user markets for ACT in the GMS.

### 4.3 Episode 3: the formation of actual end-user markets for ACT in the GMS (early 2000s to mid-2010s)

#### 4.3.1 Historical overview

**Fig. 7:**
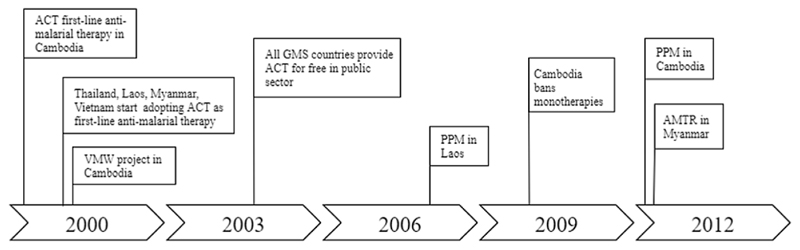
Timeline of the formation of end-user markets for ACT in the GMS (Episode 3).

At the beginning of this millennium, multidrug resistance to conventional monotherapies had become widespread in the GMS and it was clear that the region would have to rely on the deployment of ACT. In 2000, Cambodia was the first country to adopt an ACT of co-administered artesunate plus mefloquine as national first-line therapy (Novotny et al., 2016). Later that year, Thailand did the same by stipulating the use of artesunate plus mefloquine for *all* malaria patients within the country (ACTwatch, 2016a). Between 2001 and 2003, Vietnam, Laos and Myanmar followed by including ACT in their national treatment guidelines. Each country mobilized resources to subsidize ACT in public sector facilities and from 2003 onwards, this was supplemented with global-level GFATM subsidies (WHO, 2017a). Furthermore, the health ministries of each GMS country engaged in promotional activities to inform the population about the intended transition to ACT (ACTwatch, 2016a, 2015a, 2015b; Novotny et al., 2016; Yeung et al., 2011). However, despite the clinical superiority of ACT compared to failing alternatives, the GMS was slow in the uptake of ACT (ACTwatch, 2016b). For years, many patients remained being treated with outdated, substandard or even counterfeit medicines (Dondorp et al., 2004). Moreover, artemisinin monotherapies remained being widely deployed in the region, despite the increased risk of recrudescence and accelerated artemisinin resistance.

A number of underlying dynamics have been associated with the problematic uptake of ACT in the GMS. One major obstacle was that malaria in the GMS is mostly prevalent amongst hard to reach populations in remote border areas, such as forest workers and labor migrants (Cui et al., 2012; McMichael and Healy, 2017). These populations are often beyond the scope of public health systems. Although conventional monotherapies were so cheap and widely available that they even reached most of these remote populations, this was not the case for ACT in the early days (Arrow et al., 2004). One intervention that turned out to be effective in improving ACT uptake amongst remote population were community-based health initiatives such as Village Malaria Worker (VMW) programs (Yeung et al., 2008). The goal of these programs was to integrate access to ACT in the broader goal of improving health coverage amongst hard-to-reach communities with limited access to health services. This was achieved by assigning community members with the VMW status and providing them with training and commodities, including diagnostic tools and ACT. Thailand was the first country to start a VMW project in 1995 and Cambodia initiated a similar project in 2001. In the following years, Laos, Vietnam and Myanmar started their own VMW programs. Community-based health programs are nowadays institutionalized in public health services in the GMS and they are considered to be a key intervention in the battle against malaria (Dondorp et al., 2017).

Another problem regarding ACT uptake was that institutional arrangements such as the GFATM subsidies, exclusively facilitated the uptake of ACT in *public sector* channels. At the same time, *private sector* channels dominated the supply of antimalarial commodities in many GMS settings, in particular in Cambodia, Myanmar and Laos (WHO, 2017a). These private sector channels remained being neglected by most arrangements. As a result, only few people in the GMS who went to private sector facilities actually received ACT (Phok et al., 2017). The private sector challenges were even further amplified by the high amount of out-of-pocket expenditures in the GMS, which enhanced the demand for low-cost alternative therapies. Global- and national-level arrangements would eventually and gradually improve availability and affordability of ACT in the private sector, including the AMFm/CPM subsidies which have been associated with varying degrees of success (Interview 2, Davis et al., 2013; Tougher et al., 2017).

The health ministries of the GMS countries engaged in regulatory activities to improve private sector compliance. Thailand and Vietnam, two countries in which private sector supply of medicines had traditionally been limited, exclusively mandated malaria treatment to the public sector, prohibiting the deployment of antimalarial therapies in the private sector (Interview 3 & 5). This way, it would be more feasible to ensure guideline compliance and adequate ACT prescription. However, the architecture of the healthcare systems in Cambodia, Laos and Myanmar called for alternative solutions. In those countries, significant proportions of the population relied on healthcare through private-sector channels (WHO, 2017a). Prohibiting the sale of antimalarial therapies in private-sector facilities would inevitably mean that those patients would miss out on appropriate treatment (Phok et al., 2017). Hence, rather than following the path of Thailand and Vietnam, complementary activities were initiated to improve compliance to ACT in the private sectors of Cambodia, Laos and Myanmar. One such initiative was the establishment of a Public-Private Mix (PPM) program in Laos, later followed by a similar project in Cambodia. The goal of these PPM programs was to enhance malaria treatment in the private sectors by providing quality commodities such as ACT therapies and diagnostic tests from the public sector, and by improving compliance to national treatment guidelines through supervision and educational activities (Novotny et al., 2016; Simmalavong et al., 2017; Yeung et al., 2011). A similar initiative was later established in Myanmar as the Artemisinin Monotherapy Replacement (AMTR) project. This project specifically aimed to discourage the deployment of artemisinin monotherapies in private sector facilities, a practice that persisted especially in Myanmar.

Despite these measures, private-sector adherence to ACT deployment remained limited in many settings in the GMS, and more drastic regulatory measures were required. In 2009, after general efforts in strengthening the public healthcare system, the Cambodian government decided to officially ban the prescription of artemisinin monotherapies and to legally act upon the deployment of any non-ACT for the treatment of malaria. This included sending law enforcers disguised as normal clients to retailing outlets to order anti-malarial therapies. Retailers and prescribers that administered inadequate therapies would receive penalties and even risked to be shut down (Novotny et al., 2016; Yeung et al., 2011). Now, the public sector is the only channel that is mandated to test and treat malaria in Cambodia, similar to Thailand and Vietnam (Interview 5). Laos and Myanmar have also engaged in regulatory measures that aim to improve private sector adherence to ACT for the treatment of malaria (Phok et al., 2017; Simmalavong et al., 2017). However, challenges do still persist towards improving the private sector uptake of ACT in those countries (ACTwatch, 2016b).

#### 4.3.2 Analysis of episode 3

**Fig. 8:**
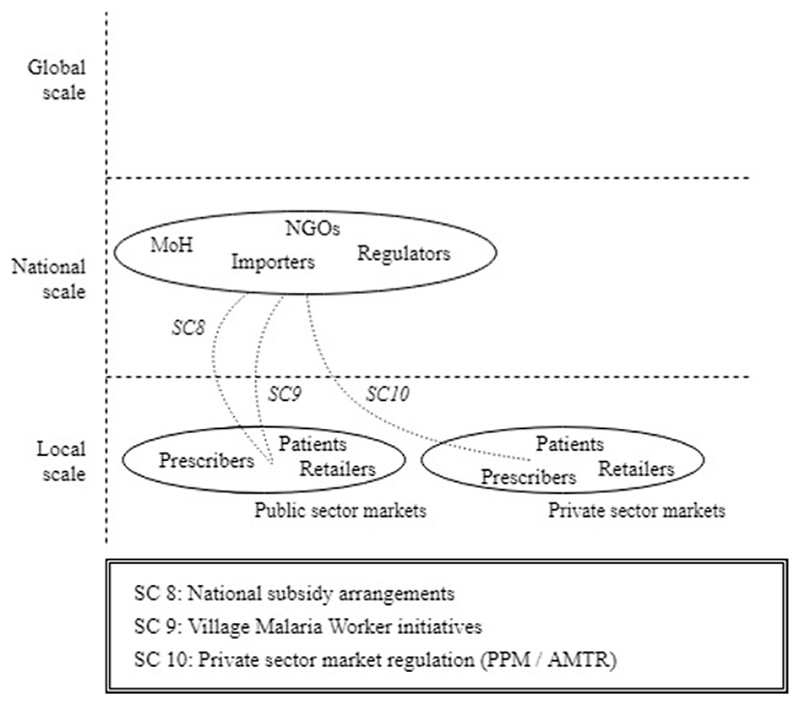
Emerging structural couplings in Episode 3.

The third episode revealed how the earlier advancements in the innovation system enabled the formation of actual end-user markets for ACT in the GMS. The episode departed when Cambodia adopted ACT as national first-line antimalarial therapy, soon followed by the other GMS countries [F4, F7]. At this point, only small niche ACT markets had been created (Episode 1) and the supportive global landscape was still under development (Episode 2). However, confronted with the high burden of drug-resistant malaria and encouraged by the explicit WHO support, the GMS countries started to pursue the formation of actual ACT markets [F5]. We observe how three structural couplings [SC8-SC10] emerged between the national scale and local market formation activities (Fig. 8).

A first structural coupling [SC8] was attained through the country-level subsidy programs that enhanced access to affordable ACT in the public sectors [F5 MF1]. The allocation of these national funds (later supplemented with GFATM subsidies) [F6], were initiated in parallel with the inclusion of ACT in national guidelines [F7, F4] and the promotional activities by national governments [F4]. The narrative then revealed that market transactions [F5 MF2] in the public sector market segments organically followed from the supportive institutional conditions and the distribution infrastructures.

The second structural coupling [SC9] was attained in the form of the Village Malaria Worker (VMW) programs which aimed to enhance ACT availability by hard-to-reach populations. These populations were initially beyond the scope of health services, but became incorporated in public sector distribution channels through the VMW initiatives [F5 MF1] which provided them with ACT and other health commodities [F5 MF2].

The final structural coupling [SC10] in this episode emerged through the regulatory and programmatic initiatives that aimed to facilitate ACT uptake in the private sectors [F5 MF1]. In these private-sector channels, the institutional environment was initially not supportive for ACT deployment and further efforts were required for market transactions to-be-formed. The narrative showed that regulatory programs were employed and adapted to local contexts to enhance the private sector uptake of ACT. Examples of these initiatives were the Public-Private Mix (PPM) programs in Cambodia and Laos and the Artemisinin Monotherapy Replacement (AMTR) project in Myanmar [F4, F5 MF2]. Thailand and Vietnam, and later also Cambodia, took more drastic regulatory measures by exclusively mandating public-sector facilities to test and treat malaria [F4, F7]. What becomes clear is that, although market formation subprocesses predominantly took place at the local scale, they resided on activities and developments at the national and global scales.

## 5 Discussion

We explored the antimalarial drug transition to ACT in the GMS in order to understand how markets are being formed at multiple geographical scales and locations. The study makes four contributions that we will discuss in-depth here. First, the study adds to the technological innovation system literature by investigating market formation as being interlinked to the other system functions. Three episodes of ACT market formation were distinguished. The first episode revealed the role of public institutes, academia and partnerships in early innovation system development. We observed how reinforcing patterns involving knowledge development, knowledge diffusion, and guidance of the search activities led to the optimal prescription form of ACT. Similar reinforcing motors of change were earlier found in other areas, such as in the sustainable mobility transition (Suurs, 2009) and the plant-based protein transition (Tziva et al., 2020). The second episode demonstrated how financial and entrepreneurial barriers undermined the formation of ACT markets and how collective efforts by globally operating organizations enabled overcoming these barriers. The third episode illustrated how these advancements led to the formation of actual ACT end-user markets in the GMS.

The study applied the three market formation subprocesses as proposed by Dewald & Truffer (2012). The first two subprocesses, market segmentation and market transaction formation, were represented by the development of public-, and private-sector distribution channels for ACTs and the related exchange relationships. The third subprocess concerns the active role of end-users in developing deployment practices and mostly took place during the early stages of innovation system development. In these early stages, clinical researchers actively participated in market formation in their search for solutions to treat drug-resistant malaria infections. The acquired insights eventually led to the optimal prescription form of ACT as well as the development of fixed-dose end-products to encourage adequate deployment. In later stages of market formation, the involvement of prescribers and patients became less prominent and market formation was rather dictated by regulatory and financial arrangements. Although there is evidence for active early user involvement in the pharmaceutical sector (DeMonaco et al., 2006) and in medical innovations in low-income countries (Hartley et al., 2019), patients and prescribers in this global health transition were mostly bound by guidelines and regulations in the later stages of market formation (Sheikh and Uplekar, 2016; Sullivan and Ben Amor, 2016).

Our conceptual approach aligns with the current literature on markets in transitions studies. We go beyond markets as pre-existing spaces in which supply and demand of products and services meet (Bleda and Chicot, 2020; Vargo et al., 2017). Instead, we perceive markets as dynamic entities and subject to the activities of several stakeholders, ranging from firms to public organizations (Fligstein and Dauter, 2007; Nenonen et al., 2019). The markets to-be-formed consist of several components that cover exchange and transactions practices (Ottosson et al., 2020), institutions (Moors et al., 2018) and narratives that legitimize its existence and its boundaries (Navis and Glynn, 2010; Santos and Eisenhardt, 2009), as was revealed by the guidance of the search and legitimacy creation activities we found throughout the three episodes. Our findings contribute to deepening the understanding of market formation processes in the context of innovation systems and transitions, which is all the more prominent as many sustainable innovations enter a phase in which they are ready to scale-up (Boon et al., 2020; Hyysalo et al., 2018). As such, we broaden the notion of diffusion in the context of transitions.

A second extension to the current literature lies in investigating which actors and institutions play a role in the under-explored field of global health transitions (Kukk et al., 2016; van Welie et al., 2019). We focused on a global trend that requires attention to sustainable transitions in global health: the recurrent emergence of drug resistance to therapies for poverty-related infectious diseases. Understanding how markets for new therapies are created is required for facilitating transitions towards more sustainable treatment regimens. Previous scholars have emphasized the critical role of country-level dynamics in the formation of markets for innovative therapies (Kukk et al., 2016; Moors et al., 2018). Those studies argue that endeavors such as regulatory procedures, market approval and reimbursements decisions are typically organized at the national levels. In the present study, we show that the formation of antimalarial drug markets also relies on dynamics at the local and transnational levels. Taking an innovation systems perspective, we perceived these activities of drug development, regulation, distribution and utilization as being interdependent and mutually contributing to the formation of end-user markets. We additionally demonstrate that these global health transition activities play out at different geographical scales and locations.

The multi-scalar nature of global health transitions leads to a third contribution. Previous scholars have emphasized the importance of local actor networks and institutions in market formation processes (Dewald and Truffer, 2011; Martiskainen and Kivimaa, 2019; Matschoss and Heiskanen, 2018). Our study extends this with a transnational orientation that includes multiple geographical scales and locations. In doing so, this research builds on the work of Binz & Truffer (2017), who introduced the notion of Global Innovation Systems (GIS) and argued that there is a need for improved understanding of structural couplings between geographical dispersed subsystems. The three episodes showed ACT as an emerging technology first spread from country to country (Episode 1), after which a supportive global landscape for ACT development and deployment was created through collective efforts by transnationally operating organizations and institutes (Episode 2), which was then followed by national and local market creation activities (Episode 3). We observed how the formation of public-sector markets in the GMS turned out to be relatively straightforward after the inclusion of ACT in global and national treatment guidelines. This was, however, not yet the case with private-sector markets, which required complementary institutional, regulatory and financial arrangements.

Structural couplings were crucial in enabling interactions between the geographical scales and locations. Our study demonstrated that structural couplings in a global health transition span across different geographical scales (global, national, local) and can take several forms, including product-development partnerships (DNDi, MMV), regulatory arrangements (WHO pre-qualification scheme), subsidy programs (GFATM/AMFm subsidies), and programmatic initiatives (VMW, PPM). These structural couplings are essential for the functioning of the global innovation system and they enabled the formation of dispersed end-user markets for ACT in the GMS. The obtained insights in structural couplings can provide lessons for future sustainability transitions at multiple scales and locations.

Fourth, the insights of this study are relevant in the context of much needed understanding of science, technology and innovation dynamics in times of global health emergencies such as COVID-19 and anti-microbial resistance. A worrying development in the field of malaria is that parasites in the GMS have started to develop resistance to artemisinin and partner drug combinations. Hence, new therapies and associated markets are again urgently required (van der Pluijm et al., 2021; White, 2019). The present study provides lessons learned that can be used to inform the formation of markets for next-generation therapies. These include the power of bottom-up initiatives by clinical researchers in optimizing deployment practices, the value of product-development partnerships in translating medical-technological potential into actual end-products, and the potential of institutional learning and sharing best practices amongst countries to optimize market transactions.

In this study, we focused on the market formation of ACT in the GMS because this region is considered to be the epicenter of drugresistant malaria. Future transition studies in global health should extend this work by focusing on drug transitions in other geographic regions and for other poverty-related diseases. This would add to the empirical evidence base of transition studies in global health and could inform policy makers in strategic decision making towards more sustainable treatment regimens.

## 6 Conclusion

This article demonstrated how markets for Artemisinin Combination Therapies (ACT) were formed at multiple geographical scales and locations. We showed how the pressure of drug resistance led to the discovery of artemisinin, a new class of antimalarial therapies, and how public institutes, academia and partnerships contributed to the early development of the innovation system. Market formation activities became heavily interlinked with other systemic activities such as knowledge development and legitimacy creation. We revealed how globally operating organizations and institutes created a supportive global landscape for ACT development and deployment and how these advancements led to the formation of ACT markets in the GMS. We demonstrated how structural couplings were attained between innovation system components at the global, national and local scales. These structural couplings emerged in several forms, including funding mechanisms, product-development partnerships, and regulatory arrangements and they enabled the formation of end-user markets for ACT in the GMS. We conclude that market formation activities in a global health transition are distributed along global, national and local scales and that they are facilitated through the attainment of structural couplings. The obtained insights are particularly relevant in times of global health emergencies which require new technological solutions and associated end-user markets.

## Supplementary Material

Appendix

## Figures and Tables

**Table 1:** Description of system functions and market formation subprocesses for innovative therapies.

**F1. Entrepreneurial activities:**	Activities to discover active drug compounds and to translate these active drug compounds into actual end-products (either through commercial or non-profit programs)
**F2. Knowledge development:**	Activities to obtain data and information on the innovative therapy. This includes studies on the efficacy, safety and tolerability and studies to optimize production, dosing and prescription behavior
**F3. Knowledge diffusion:**	The dissemination of data and information about the innovative therapy through e.g. conferences, seminars, publications and reports
**F4. Guidance of the search:**	Activities that contribute to the visibility of the innovative therapy compared to alternative treatment options, and activities that provide guidance for the further innovation processes
**F5. Market formation:**	
MF1. Market1 segmentation:	The establishment of market substructures and distribution channels for specific product-, or end-user categories of the innovative therapy
MF2. Market transactions:	The formation of exchange relationships between suppliers and users of the innovative therapy
MF3. End-user profiles:	The active role of end-users, i.e. patients and prescribers, in developing preference structures and deployment practices for the innovative therapy
**F6. Resource mobilization:**	Activities to obtain the required financial and human resources to facilitate the development, production and uptake of the innovative therapy
**F7. Creation of legitimacy:**	Efforts that contribute to the perceived legitimacy of the innovative therapy by other system actors

**Table 2:** List of interview respondents.

ID	Affiliation of respondent	Date	Mode
Interview 1	Representative of pharmaceutical industry	25–1–2019	In person
Interview 2	Country-level malaria policy representative Cambodia	25–1–2019	In person
Interview 3	Country-level malaria policy representative Vietnam	28–5–2020	Digital
Interview 4	Country-level malaria policy representative Thailand	4–6–2020	Digital
Interview 5	Regional malaria specialist Greater Mekong Subregion	11–6–2020	Digital
Interview 6	Principal malaria researcher	23–4–2020	In person

**Table 3:** Operationalized indicators.

Concept	Indicators	Example of event
**F1. Entrepreneurial activities:**	Exploratory research projects Innovative drug development projects Generic drug development projects	Project 523 to identify new antimalarial drug compounds Novartis/ Kunming collaboration to develop Coartem® Engagement in ACT by generic producers
**F2. Knowledge development:**	(Pre-)clinical studies Process optimization studies	Clinical studies with ACT at the Thai-Myanmar border Improving methods of extracting the artemisinin derivatives
**F3. Knowledge diffusion:**	Conferences / seminars (Scientific) publications Reports	WHO expert consultation meetings in 1998 - 2001 QACRG publication Published reports on ACT deployment
**F4. Guidance of the search:**	Policy guidance Standard setting Promotional activities	Inclusion of ACT in WHO malaria treatment guidelines Deployment of fixed-dose ACT Promotion of ACT on billboards, tv-campaigns
**F5. Market formation:**		
MF1. Market segmentation	Market substructures, distribution channels	Integration of ACT in public sector supply chains
MF2. Market transactions	Exchange relationships	Deployment of ACT through community health programs
MF3. End-user profiles	Preference structures, deployment practices	Experimentation during early stages of deployment
**F6. Resource mobilization:**	Financial investments Subsidy arrangements Human/technical resources	Allocation of national funds to implement ACT Allocation of GFATM procurement subsidies Knowledge transfer by pharmaceutical companies
**F7. Creation of legitimacy:**	Market regulation Regulatory approval External legitimacy creation	Withdrawal of artemisinin monotherapies from the market Registration and market approval of ACTs ACT deployment by Médecins Sans Frontières

## Footnotes

1 WHO = World Health Organization

2 GFATM = Global Fund to fight Aids, Tuberculosis and Malaria

3 MSF = Médecins Sans Frontières

4 USAID = United States Agency for International Development

5 MMV = Medicines for Malaria Venture

6 DNDi = Drugs for Neglected Diseases initiative
