# Supplementary material for: Market Formation in a Global Health Transition
Source: Environ Innov Soc Transit. Author manuscript; Available in PMC 2022 Jan 31. (PMC7612298; doi:10.1016/j.eist.2021.05.003)
Supplement: Appendix [file EMS140695-supplement-Appendix.pdf]

Appendix A

In this methods appendix we explain the six methodological steps of the study.

Step 1: Structural analysis

We made an overview of the structure of the innovation system of antimalarial therapies by mapping the key actors, networks and institutions. Data were collected by reviewing scientific articles, gray literature and informal talks with malaria experts and other global health representatives. gray literature included institutional websites, reports and news articles that were subjected to the development and implementation and utilization of ACT.

Step 2: Event timeline

A timeline was constructed which included events in the transition from conventional monotherapies to ACT, with an emphasis on market formation in the GMS. Data were collected by using the same sources as the structural analysis plus a literature review and expert interviews.

Literature review: The literature review comprised the collection and analysis of both peer-reviewed and gray literature. Peer-reviewed literature was accessed through the search engines Web of Science and Google Scholar, using search queries that aimed to retrieve all literature subjected to the transition to ACT. gray literature was accessed through using similar search terminologies in the search engine Google, and by performing targeted searches at institutional websites. Selected gray literature included policy reports, institutional press releases, and (research) articles at websites of global health organizations.

The main goal of the literature review was to identify the main events that affected the transition from conventional monotherapies to ACT and in particular the formation of markets in the GMS. Therefore, the titles, summaries and abstracts of the obtained literature were examined. If the document was considered relevant for the purpose of the study, the full content was assessed. Events that either positively or negatively influenced the transition to ACT in the GMS plus the corresponding dates were extracted from the selected literature and listed in a database. The same literature was also used to gain contextual insights for the narrative beyond the mere identification of events. The original event database contained the following columns: date, event, background, sources.

Expert interviews: Respondents for interviews were purposefully selected, based on their affiliation and area of expertise. Semi-structured interviews were conducted. Interview guides were developed and customized for each respondent. We asked general

questions about the stakeholders, networks and institutions that had been involved in the transition from conventional monotherapies to ACT. Furthermore, experts were asked to identify and confirm events that led to the formation of ACT markets in the GMS and to provide contextual background to these events. All interviews were tape recorded with consent of the interviewee and transcripts were made directly after each interview.

Step 3: Coding of theoretical constructs

Each event was allocated to one of the theoretical constructs according to the descriptions in Table 1 by using the operationalized indicators in Table 3. Codes were attributed to each event and included in the database. FdH coded all events, WB and EM verified events to increase the validity of the study. Differences in interpretation were discussed until consensus was reached.

Step 4: Writing of narrative

Based on the coded timeline of events, the corresponding dates, and the contextual comments, a narrative was constructed. The narrative followed the three major episodes of the formation of ACT markets in the GMS that had emerged from the collected data. The goal of the narrative was to map market formation dynamics over time. The narrative included references to the theoretical constructs that were allocated in step 3. This enabled the identification of functional patterns and the related transition dynamics.

Step 5: Identification of multi-scalar transition dynamics and structural couplings

We plotted diagrams to distinguish actors, functional dynamics, market formation processes and structural couplings at local, national and global scales, as well as their interrelations. These plots enabled us to comprehend the multi-scalarity of the transition processes and to identify the structural couplings between the geographically dispersed subsystems. Structural couplings were identified by using the definition that was provided in the theory section as a heuristic. The allocation of structural couplings was again discussed amongst the research team.

Step 6: Analysis of the episodes

For each of the three episodes, we wrote a case interpretation, in which we analyzed the multi-scalar transition dynamics. These interpretation sections contained an analysis of the functional dynamics of the historic overview using the coded events and the narrative. The analyses focused on the functional dynamics and the multi-scalarity of the transition from conventional monotherapies to ACT. The analysis of each episode was approached through a market formation lens, i.e. by asking how did the sequence of events, the functional dynamics and the attainment of structural couplings contributed to the formation of ACT markets in the GMS?

Finally, we created figures to illustrate the emerging structural couplings in each episode (See Figs. 4, 6 and 8).
